# BioPortal: an open community resource for sharing, searching, and utilizing biomedical ontologies

**DOI:** 10.1093/nar/gkaf402

**Published:** 2025-05-13

**Authors:** Jennifer Vendetti, Nomi L Harris, Michael V Dorf, Alex Skrenchuk, J Harry Caufield, Rafael S Gonçalves, John B Graybeal, Harshad Hegde, Timothy Redmond, Christopher J Mungall, Mark A Musen

**Affiliations:** Center for Biomedical Informatics Research, Stanford University, Palo Alto, CA 94304, United States; Environmental Genomics and Systems Biology, Lawrence Berkeley National Laboratory, Berkeley, CA 94720, United States; Center for Biomedical Informatics Research, Stanford University, Palo Alto, CA 94304, United States; Center for Biomedical Informatics Research, Stanford University, Palo Alto, CA 94304, United States; Environmental Genomics and Systems Biology, Lawrence Berkeley National Laboratory, Berkeley, CA 94720, United States; Center for Biomedical Informatics Research, Stanford University, Palo Alto, CA 94304, United States; Center for Biomedical Informatics Research, Stanford University, Palo Alto, CA 94304, United States; Environmental Genomics and Systems Biology, Lawrence Berkeley National Laboratory, Berkeley, CA 94720, United States; Center for Biomedical Informatics Research, Stanford University, Palo Alto, CA 94304, United States; Environmental Genomics and Systems Biology, Lawrence Berkeley National Laboratory, Berkeley, CA 94720, United States; Center for Biomedical Informatics Research, Stanford University, Palo Alto, CA 94304, United States

## Abstract

BioPortal (https://bioportal.bioontology.org) is the world’s most comprehensive repository of biomedical ontologies. It provides infrastructure for finding, sharing, searching, and utilizing biomedical ontologies. Launched in 2005, BioPortal now includes 1549 ontologies (1182 of them public). Its open, freely accessible website enables anyone (i) to browse the ontology library, (ii) to search for terms across ontologies, (iii) to browse mappings between terms, (iv) to see popularity ratings and recommendations on which ontologies are most relevant to their use cases, (v) to annotate text with ontology terms, (vi) to submit an ontology, and (vii) to request ontology changes. The library of ontologies can be accessed programmatically via a REST application programming interface (API). Recent enhancements include a BioPortal knowledge graph that integrates knowledge from multiple ontologies; a unified data model for interoperability with other knowledge sources; ontology popularity ratings and recommendations for relevant ontologies; and the ability to request ontology changes via a simple user interface that automatically converts user change requests to GitHub Pull Requests that specify the edits that will be made to the ontology upon approval.

## Graphical Abstract

**Graphical Abstract:**
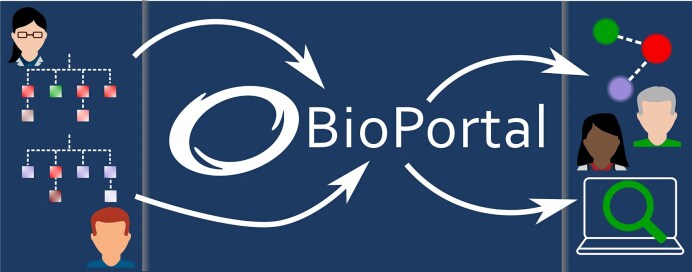

## Introduction

### What are ontologies?

BioPortal is a repository for ontologies—standardized, computable representations of the entities in some discipline that enable precise organization, curation, and integration of data across scientific domains [1]. Ontologies serve as fundamental infrastructure for modern information systems, and are widely used across the biological and biomedical sciences.

### What is BioPortal?

Throughout its existence, BioPortal’s vision has been that all biomedical knowledge and data should be disseminated using principled ontologies such that the knowledge and data are semantically interoperable and can be discovered and used to advance biomedical science. BioPortal now houses 1549 biomedical ontologies (1182 of them public) that collectively include 15 293 440 terms and over 100 million cross-ontology mappings. Its open, freely accessible user interface (UI) offers flexible options for browsing, searching, submitting, and changing ontologies. Additionally, BioPortal provides programmatic access via a powerful application programming interface (API) that facilitates extensive data interaction and integration with external systems. BioPortal is free and open to all users and there is no login requirement for browsing and searching ontologies. A free account is required to submit an ontology or to request ontology changes.

### History of BioPortal

The BioPortal project was initiated in 2005 by the National Center for Biomedical Ontology (NCBO), which was one of the NIH’s National Centers for Biomedical Computing funded under the NIH Roadmap Initiative. The NCBO aimed to bring together the bio-ontologies community and the Semantic Web community in the development of BioPortal, the first open repository of all publicly available biomedical ontologies. The first public release of the BioPortal website was in 2008; the API was made available in 2013 [2]. New funding from the NIH Data Repository and Knowledge Base initiative has allowed us to maintain and improve BioPortal, creating a BioPortal knowledge graph (KG) and implementing a semi-automated process to act on ontology changes requested by users.

### Metrics and growth

BioPortal serves a broad community of researchers with a substantial user base. Since its launch in 2008, BioPortal has continued to expand in popularity. As of January 2025, it has over 18 000 registered users, plus many more unregistered daily visitors. Each month, it receives 100 000 page views and 100 million API calls. Active usage includes activity from 1173 daily active users, 6848 weekly users, and 31 276 monthly users. More than 700 000 unique IP addresses access BioPortal monthly. Table 1 provides further details.

**Table 1.** BioPortal metrics as of 3 March 2025

Usage metric	Number
Registered users	18 487
Website active users (daily/monthly)	1173/day; 31 276/month
Page views per month	103 165
API calls per month	159 110 549
Unique IP addresses per month	771 012
**Storage metric**	**Number**
Ontologies (public)	1182
Ontologies (private)	367
Total ontologies	1549
Ontology classes stored	15 293 440
Ontology properties stored	36 286
Ontology mappings	101 923 217
Ontologies available as graph	1050

The first table shows usage metrics; the second shows metrics about the ontologies and mappings stored in BioPortal.

Like many web resources, BioPortal has seen an increase in activity due to the rise of artificial intelligence (AI) scrapers, which often ignore robots.txt, and we are looking into approaches to reduce the impact of these on users.

### Capabilities

In this section, we briefly describe the user-facing capabilities of the BioPortal web server. More details can be found in our user documentation at https://www.bioontology.org/wiki/BioPortal_Help.

### Search, browse, and choose

BioPortal offers a range of features for browsing, searching, and receiving recommendations for ontologies. The BioPortal landing page supports several ways to look for ontologies and terms. To find a particular ontology quickly, a user can start typing part of its name in the search box under “Find an ontology;” a dropdown will appear showing ontologies with names that include that string. The “Browse ontologies” button opens a dropdown that allows users to browse all ontologies in BioPortal. Alternatively, users can choose the name of a group of related ontologies [such as ontologies in the OBO Foundry or in the Unified Medical Language System (UMLS)] and then browse the particular collection. The landing page also lists and links to the five most popular ontologies (by number of visits). At the top of the page there are links to other ways to access the information in BioPortal (Ontologies, Search, Annotator, Recommender, and Mappings); these are described below.

### Browsing ontologies

**Figure 1.**
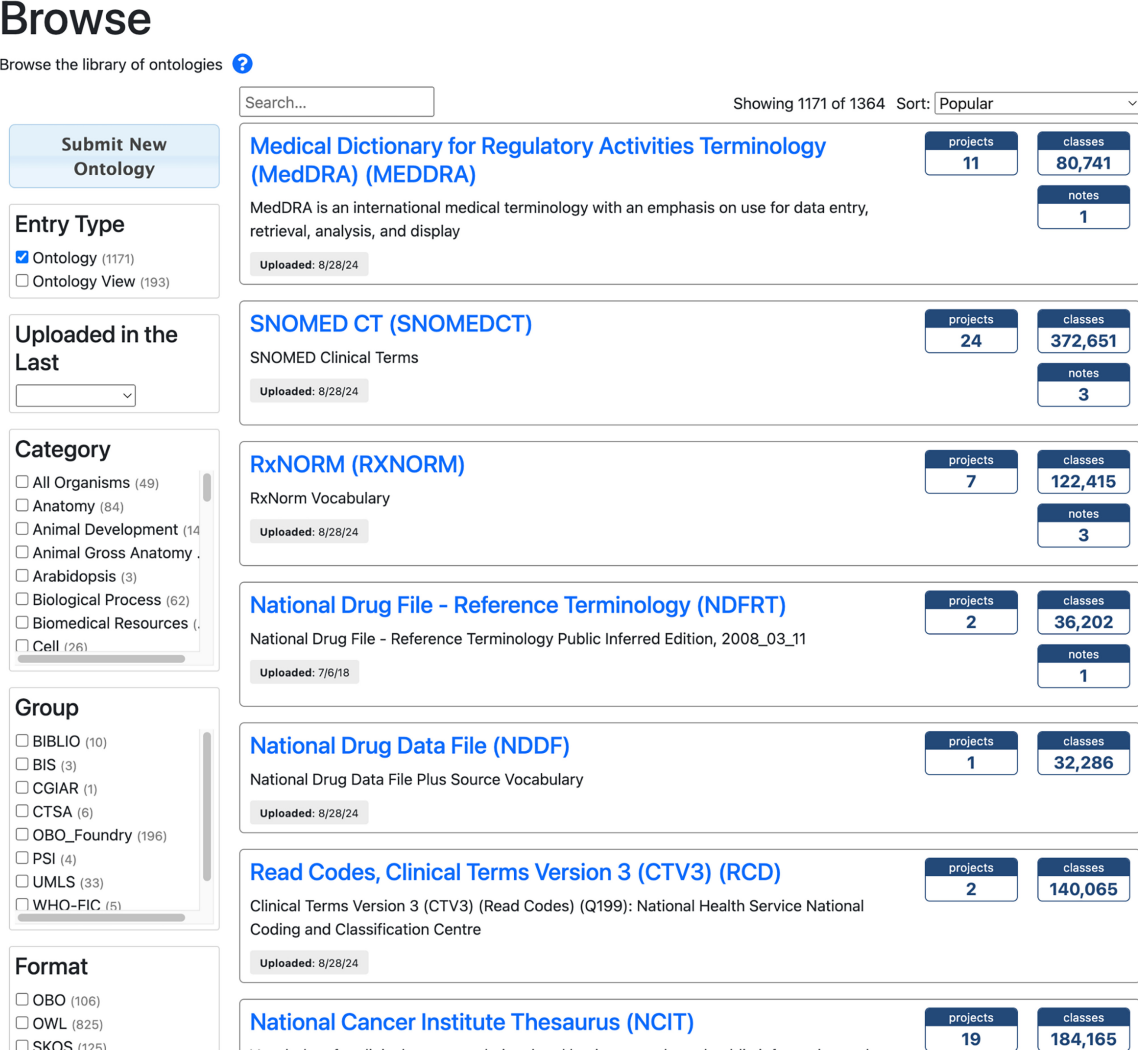
The ontology browser shows all of the ontologies in BioPortal, or in the subgroup the user selected. Each ontology listing includes hyperlinked summary statistics: number of terms (classes) in the ontology, number of notes, and BioPortal registered projects that use the ontology.

After a user chooses “All” or a collection of ontologies from the “Browse ontologies” page (https://bioportal.bioontology.org/ontologies), the relevant subset of ontologies will appear in an ontology browser page (Fig. 1). Each ontology listing on that page includes

- The name of the ontology (hyperlinked to its BioPortal ontology page)
- A brief description
- Date when it was last updated in BioPortal
- Metrics, all of which are hyperlinked to more information
  - Number of projects that use this ontology
  - Number of classes in the ontology
  - Notes (if any)

More detailed metrics and other ontology information can be found on the individual ontology pages, which are described later.

By default, the ontologies listed on the ontology browser page are sorted in descending order by popularity, which is currently measured using the number of Google Analytics page views from the previous month. Other sort options are available via the Sort menu. The checkboxes on the left side of the page enable filtering of the listed ontologies, e.g., by category (biomedical domain), by date last updated, or by format in which the ontology can be downloaded.

### Ontology pages

Each ontology in BioPortal has a summary page that serves as the ontology’s landing page in BioPortal and includes detailed metadata about the specific ontology. Icons on the top right let users download the ontology, go to the ontology project’s documentation page, or go to the publications page (if there is one) for the ontology. Ontology pages have multiple tabs, including a metadata summary, a hierarchy of all classes, any properties, community-provided notes, and mappings to other BioPortal ontologies. A Widgets tab provides quick access to reusable code for searching and visualizing the ontology.

**Figure 2.**
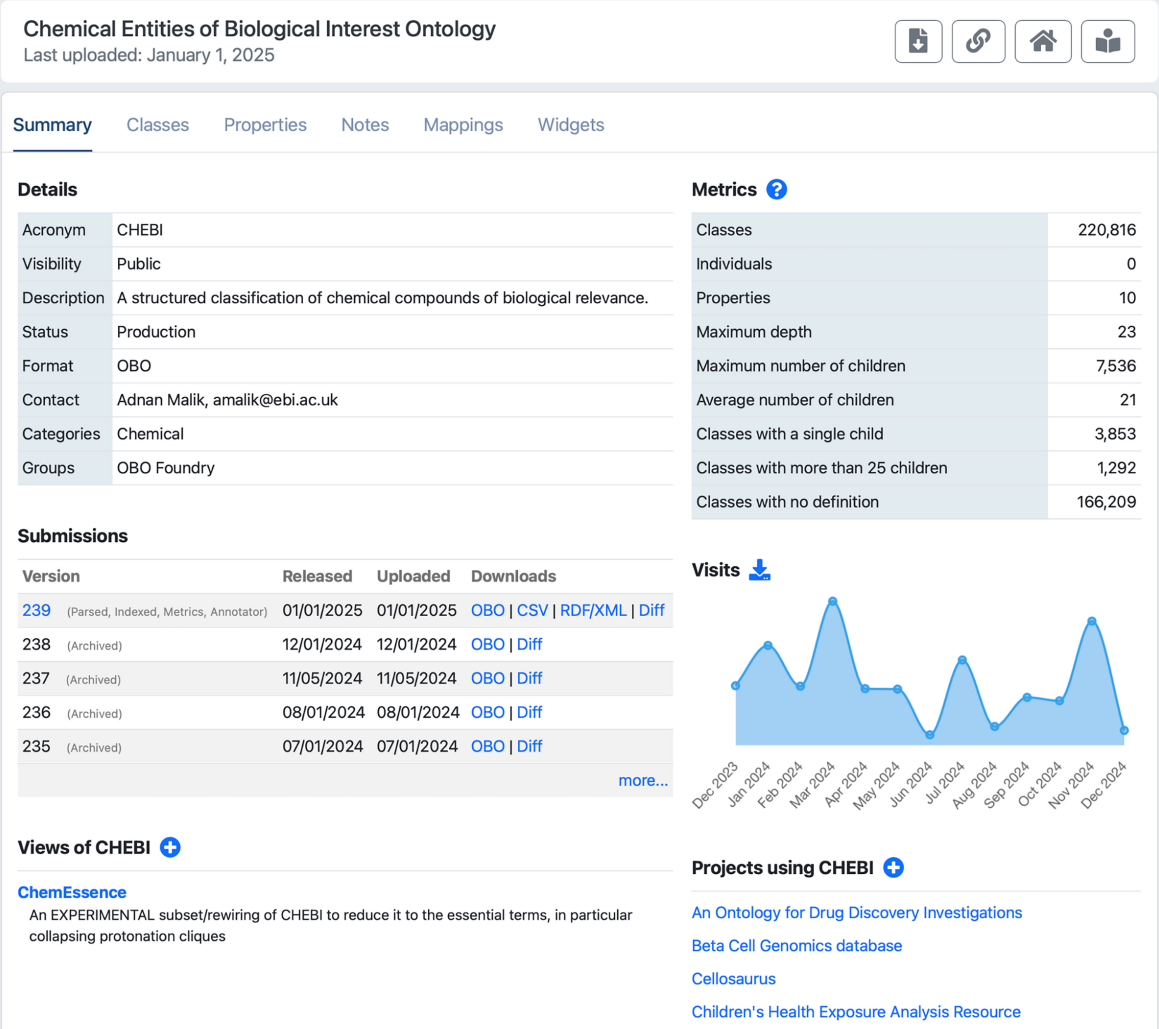
An example of an ontology summary page (this one for the Chemical Entities of Biological Interest ontology). BioPortal provides a front page for each ontology, with tabs (“Summary,” “Classes,” “Properties,” “Notes,” “Mappings,” and “Widgets”) for exploring the ontology’s content and associated resources. The “Summary” page, shown here, includes the following sections: (i) the “Details” section provides ontology metadata such as the ontology’s acronym, public visibility status, format (e.g. OWL, OBO, and SKOS), contact information, the category that best describes the ontology’s domain, and the group that it belongs to (e.g. UMLS and OBO Foundry). (ii) The “Submissions” section details version history and provides options for downloading the ontology in multiple formats like OBO, CSV, or OWL-RDF/XML. (iii) The “Views” section lists reusable subsets of the ontology. (iv) The “Metrics” section offers metrics on the ontology’s composition, including the number of classes, individuals, properties, and other relevant statistics. (v) The “Visits” section provides analytics on user interactions with the ontology, displaying metrics such as the number of recent visits and trends in usage over time. (vi) The “Projects” section lists related (ontology) projects or initiatives that use or contribute to the selected ontology.

The **Summary** tab (Fig. 2) for an ontology includes more details and metrics, as well as a table of releases (with links to download the ontology file) and a graph of visits to the ontology over the past year. Below those is a list of views (sometimes called “slims”) that represent curated subsets of the ontology, and a list of projects that use the ontology.

**Figure 3.**
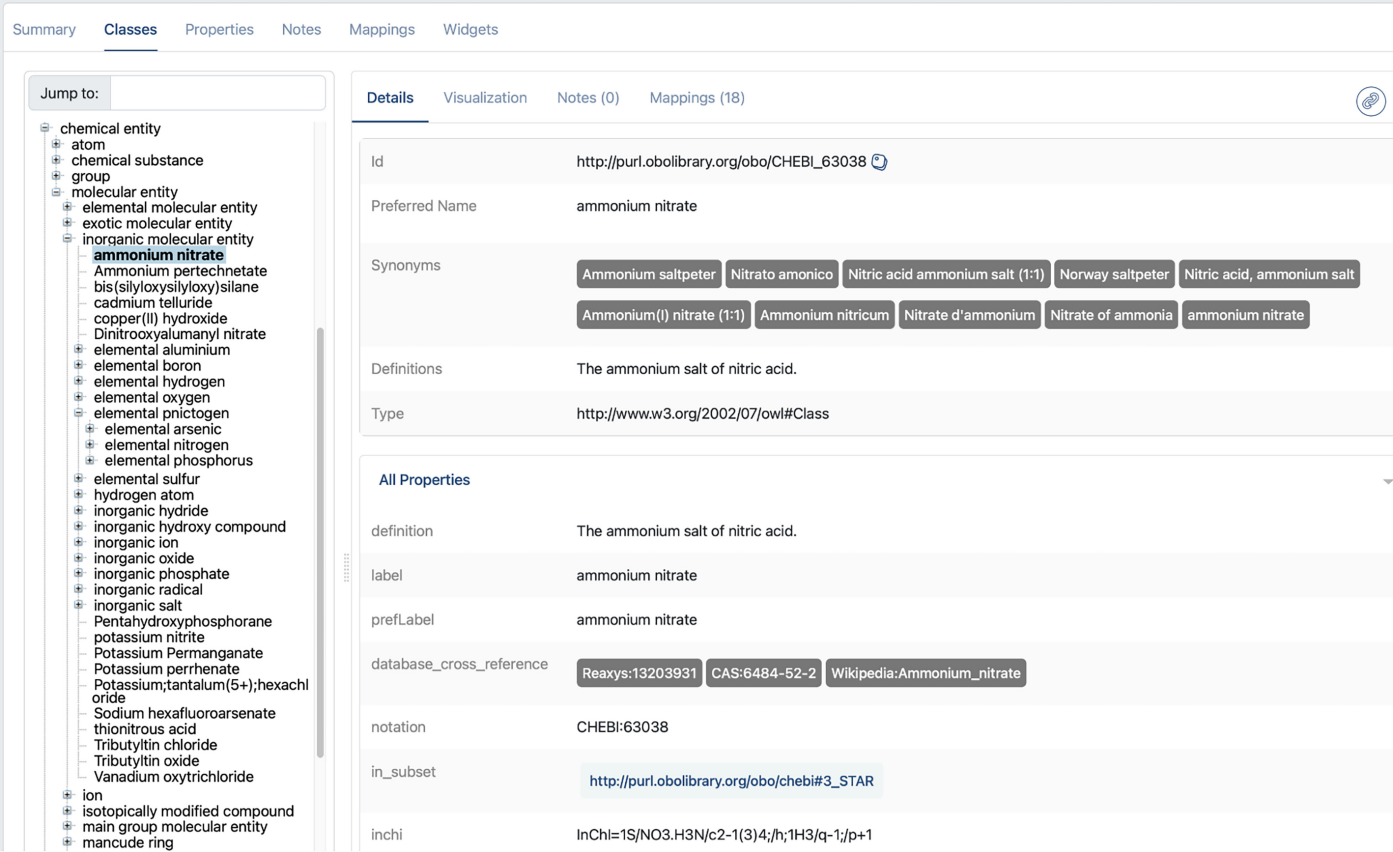
The Classes tab displays a hierarchy of classes (terms) in the ontology on the left; the details for the currently selected class appear on the right. A plus sign to the left of a class name means that it has child terms that can be opened.

The **Classes** tab (Fig. 3) lets users hierarchically browse the ontology’s classes (also called concepts). Recent enhancements to the Class display page have significantly improved the usability and readability of class information by enabling independent scrolling between the tree and details panes. Additionally, the concept details pane has been redesigned to prominently display essential information—such as the ID, Preferred Name, Synonyms, and Definitions—at the top of the page. The remaining concept properties have been consolidated into a section titled “All Properties.” This section is designed to be expandable and contractible, ensuring the term display remains compact and navigable. Synonyms are now presented in a streamlined “badge” view, which clearly highlights each synonym in a horizontal arrangement. To maintain a compact interface while providing access to more detailed information, a “See more” link has been added, allowing users to expand the view when needed.

Other views available in the Classes tab include a visualization of the ontology structure and class mappings. Mappings are associations between two or more terms in different ontologies; they typically, but not always, represent a degree of similarity between the terms. Most of the mappings in the BioPortal knowledgebase are generated automatically by the system [3]. These mappings can inform the user that, e.g., the term “agar” in CHEBI (CHEBI:2509) is equivalent to terms in other ontologies such as MESH (MESH:D000362), RxNORM (RXNORM:397), and SNOMED CT (SCT:10249006) (courtesy of lexical maps), while the CHEBI term itself is reused in several other ontologies, including FOODON (Food Ontology) and DRON. Mappings can also go beyond lexical matches: For example, the term “Raised intraocular pressure” in the Read Codes, Clinical Terms Version 3 (RCD) has a corresponding MEDDRA term “Intraocular pressure high” and maps to “GLAUCOMA” in the World Health Organization Adverse Reaction Terminology, as the latter ontology groups many related terms.

### Find a relevant ontology

BioPortal offers several ways to find an appropriate ontology for a use case. The home page provides a search box that lets users locate a class (term) of interest across all of the ontologies in BioPortal. Clicking “Advanced search” brings up more options (e.g. include only exact matches, or to search particular categories). The matches are hyperlinks that open the ontology hierarchy with the requested class selected.

Another BioPortal service for locating a relevant ontology is the Recommender (https://bioportal.bioontology.org/recommender) [4], which lets users enter an excerpt from a biomedical text or a list of keywords, and then finds the most relevant ontologies for the entered text. It determines which ontologies to recommend based on user-established weightings of characteristics such as precision, recall, ontology size, and frequency of ontology use.

### Annotator service

The related Annotator service, which also accepts text from a biomedical journal or other source, finds specific ontology terms that relate to the words in the text. An augmented Annotator service called Annotator+ (https://bioportal.bioontology.org/annotatorplus) [5] provides more advanced annotation capabilities, such as the ability to detect negation, particularly important for clinical text; e.g., in the phrase “the patient has no signs of melanoma,” the concept “melanoma” is noted to be negated. These advanced services are publicly accessible via UI and API.

### Submit an ontology

BioPortal encourages its user community to upload to the repository all relevant ontologies related to the biological or clinical sciences. To submit an ontology, the submitter must be a registered user and signed in, and the ontology must be available in one of the three most common languages: OWL (Web Ontology Language), OBO (Open Biomedical Ontologies), or SKOS (Simple Knowledge Organization System). Any standard OWL serialization format that can be parsed using the OWLAPI is accepted in BioPortal (e.g. RDF/XML and Turtle). As part of the submission process, users are asked to provide certain metadata attributes, as described in the BioPortal documentation.

By default, all ontologies in BioPortal are publicly viewable (without requiring login). If the ontology owner does not want the public to see their ontology, it can be marked as “private” during submission, with access granted to specified registered users. Although it may seem counterintuitive to submit a private ontology to an open ontology repository, many ontology developers at commercial organizations want to be able to use BioPortal’s tools and services without exposing their ontology to the general public.

### Request changes to an ontology

Ontologies are not static; they are artifacts that evolve as our understanding of the ontology’s domain advances or our requirements for the ontology change. Community input is essential to improving ontologies so that they provide ever more accurate and useful structured information about the relevant domain. Changes to ontologies can take many forms, including adding or deleting terms, merging terms, changing definitions, adding synonyms, and more.

The traditional process of changing an ontology is time-consuming and requires considerable domain knowledge and technical expertise. Members of the ontology user community are not usually able to make these changes themselves; instead, there is a bottleneck while they wait for an ontology curator to respond to a change request. There is also no standard mechanism for communicating change requests to ontology owners. We designed the new BioPortal ontology change request service to accelerate this process. Under the hood, this facility required a standardized way to express changes in ontologies. To this end, we created KGCL (Knowledge Graph Change Language) [6]. KGCL can represent in a concise and unambiguous manner common ontology editing operations (such as modifying a label or a definition, making a term obsolete, or moving a term under another parent term).

BioPortal users are not expected to use KGCL to express their ontology change requests, however. BioPortal’s user-friendly change request UI provides access to simple forms for entering information about proposed changes to specific ontologies that have been configured to be “community-editable.” The forms request information that is relevant for the change type (e.g. for the addition of a synonym, a dropdown field allows the user to specify the type of synonym such as exact, narrow, broad, or related). This information is used by BioPortal to automatically generate an issue (also known as a ticket) in that ontology’s GitHub (a popular version control system used by many ontologies) repository describing the desired change. Each such issue includes a machine-processable string that precisely describes the requested change as a KGCL command, as well as a human-readable description. A curator for the ontology is notified about the change requests and can quickly assess these issues and approve or reject them. When one of these issues is approved by a curator, the change agent software automatically generates a GitHub action to make the requested edit to the ontology file. This process, while still relying to some extent on human expertise, is much faster, friendlier to less technical users, and less error-prone than the typical ontology-editing workflow.

### Programmatically access BioPortal functionality

Many BioPortal users access our content programmatically rather than via our UI. Currently, we handle over 159 million API requests each month (see Table 1).

We are aware of more than a hundred applications that call the BioPortal API. These include clinical decision support systems, electronic health record systems, medical coding tools, genomic data annotation platforms, biomedical text mining applications, AI-powered medical chatbots, and radiology report structuring systems. These applications benefit from the API’s ability to standardize, map, and analyze biomedical data using a vast collection of ontologies, improving interoperability, data accuracy, and decision-making in healthcare and research. These systems range from simple scripts for answering a specific question, to complex software systems that have developed large user communities. REDCap [7] is a good example of the latter: every day, thousands of clinical investigators fill out case-report forms for clinical trials using REDCap, which communicates with BioPortal each time a user needs to fill in a field using a controlled term. The CEDAR Workbench [8], a web application for modeling metadata schemas and annotate datasets and digital objects in any domain, makes extensive use of BioPortal’s semantic capabilities and API services.

Python programs can access the BioPortal API through the OntoPortal Client (https://ontoportal-client.readthedocs.io/en/latest/) or Ontology Access Kit (*oaklib*) [9] packages, both available through the Python package index.

### BioPortal KG

KGs are graph-based data structures for representing heterogeneous relationships among varied data types, including the curated knowledge within ontologies. KGs form a foundation for powerful but flexible approaches for integrating data with expert-validated domain knowledge. By enabling interoperability across ontologies and data, KGs support new ways to make inferences in biology and other domains [10].

We have built a BioPortal KG (KG-Bioportal) tool that can merge relevant selections of the BioPortal ontology collection using a common graph format and a single data model (the Biolink Model). This opens the door to answering queries that require information to be obtained from multiple sources. As of September 2024, the collection of all graph-ready ontologies in BioPortal includes about 10 million nodes (representing ontology terms) and 20 million edges (relationships between terms). Metrics about the collection, including the number of nodes and edges in each ontology, are available at https://ncbo.github.io/kg-bioportal/.

To construct KG-Bioportal, we built an open-source computational pipeline that translates ontologies from their original format into the standard KGX graph format (https://github.com/ncbo/kg-bioportal). Rather than combining all ontologies in BioPortal, the graph versions of each ontology in KG-Bioportal may be merged as needed through the KG tool’s command line interface or downloaded individually. The pipeline adds provenance metadata to each ontology that is integrated into the KG, including the version and source of the ontology. Our KG building strategy combines elements of previous approaches (e.g. semantic types from UMLS) with flexible graph assembly software infrastructure to support creation of made-for-purpose graphs with a common data model. This methodology supports creating KGs to answer specific questions by integrating semantic resources across the numerous domains covered by BioPortal. The resulting graphs are a manageable size and avoid including material unrelated to the KG’s intended use case.

This unified resource facilitates interoperability with other biomedical knowledge sources, including not just other ontologies but also a growing ecosystem of KGs and tools for interacting with them. For example, KG-Bioportal graphs can be easily combined with other data resources developed using KG-Hub practices [10] or efficiently prepared for AI applications using the GRAPE package [11]. From the user perspective, KG-Bioportal enables more integrated, powerful, and flexible querying of BioPortal ontologies. If, e.g., a researcher is interested in identifying drugs known to have a risk of causing acute kidney injury “and” their potential interactions with other drug therapies, they can benefit from drug and chemical knowledge (e.g. in RXNORM and CHEBI) that is integrated with drug–drug interaction information (e.g. from DINTO) and even general biomedical vocabulary (e.g. from NCIT). Or, if a researcher asks “what kinds of adverse interactions may occur if a patient receiving treatment for atrial fibrillation drinks grapefruit juice?”, then a focused graph can connect the FOODON term for “grapefruit food product” (FOODON:00001929) with a class in the Food Interactions with Drugs Evidence Ontology (FIDEO) defining an interaction between grapefruit and the drug dronedarone (FIDEO:000001707), link this drug to its corresponding CHEBI entry (CHEBI:50659) which is conveniently annotated as having the role “anti-arrhythmia drug”, and map the CHEBI entry to its equivalent in RxNORM (233698), where the drug can be further contextualized with tradenames (e.g. “Multaq”) and specific forms (e.g. “dronedarone 400 MG Oral Tablet”). In this way, the value of ontology-based knowledge is magnified: Conceptual relationships from different domains and disciplines are combined within a KG, so paths between otherwise distantly connected resources like FOODON and RxNORM become accessible.

### Architecture and back end

**Figure 4.**
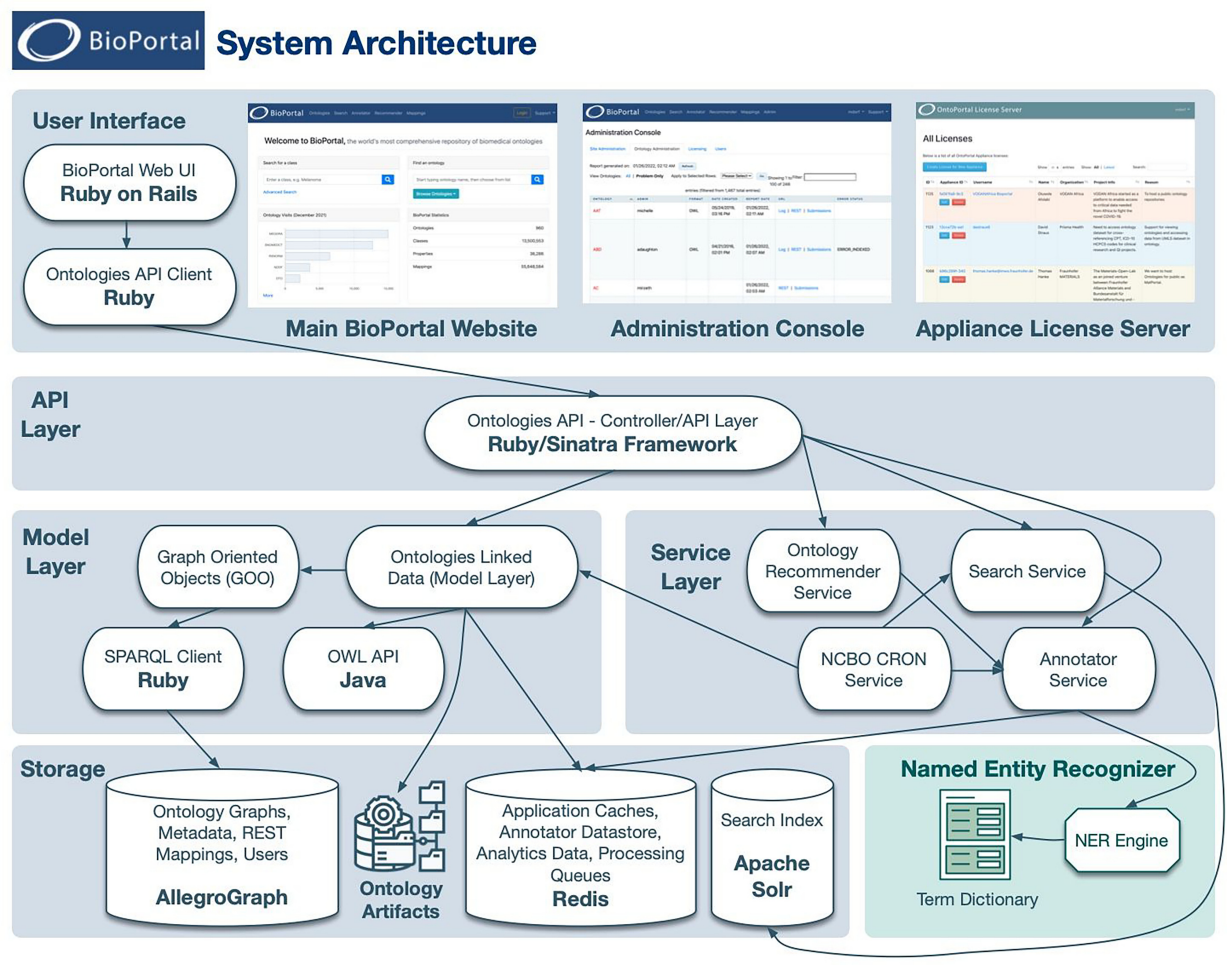
BioPortal architecture. This diagram provides a visual representation and an overview of BioPortal’s architecture, showing how the components interact within a layered framework. BioPortal’s stack consists of Ruby on Rails for web development, the Sinatra framework for API management, Java for handling ontology data, a triple-store based database (AllegroGraph), as well as a host of other supporting libraries. Redis is used for caching and session management, while Apache Solr powers the search functionality, enabling efficient, fast retrieval of ontology data. The Named Entity Recognizer engine, Mgrep, is the tool that powers the NCBO Annotator service (https://sourceforge.net/projects/multiline-grep/).

BioPortal is designed with a multilayered architecture (Fig. 4), which integrates components built using modern web technologies across several stacks to ensure robustness, scalability, and ease of use for biomedical ontology storage, retrieval, and management. The architecture incorporates several layers, including UI, API management, and data processing and storage services. This setup ensures that the platform can support both current needs and future growth.

The **UI Layer** is built using Ruby on Rails. Recent additions incorporate many modern libraries such as Turbo, Stimulus, and ViewComponent. Turbo intercepts user interactions, updating only the relevant parts of the current page with the server’s response. This allows partial updates without a full reload while preserving browser back button functionality. Complementing this capability, Stimulus, a minimalistic JavaScript framework, enhances the UI with the behavior necessary for optimal functionality, working seamlessly with Turbo and the Ruby on Rails backend. The ViewComponent framework compartmentalizes individual functionality, allowing reusable components that implement specific features to be seamlessly incorporated into any part of the BioPortal UI.

The **API Layer** functions as the communication hub between the UI and deeper service layers, using the Ruby/Sinatra framework to handle API calls and route data efficiently. This layer ensures seamless data flow and integration between the front-end and back-end services.

Within the **Service Layer**, specialized services such as the Annotator Service, Ontology Recommender Service, and NCBO CRON Service perform specific functions and automate some background tasks. These services handle everything from automated ontology recommendations to scheduled data processing, crucial for maintaining system integrity. The Service Layer utilizes the structured ontology data managed by the Model Layer, ensuring that users can efficiently search, annotate, and retrieve relevant ontology data through a well-defined and responsive API.

The **Model Layer** includes important components such as the Ontologies Linked Data for ontology management and the Graph Oriented Objects for data querying. The OWL API is used for processing the ontology content and generating RDF-based data files that get stored in a triple-store database. This layer is essential for the manipulation and retrieval of ontology data, ensuring that the system can handle complex queries and data structures.

The **Storage Layer** is crucial for managing the extensive data associated with biomedical ontologies. BioPortal is currently compatible with the AllegroGraph triple-store database and supports the latest SPARQL 1.1 query language. The storage layer encompasses components that store different types of data:

1. **Triple-store databases**: These databases are designed to handle the complex, highly interconnected data typical of ontological structures. Triple-store repositories store data in a format consisting of subject-predicate-object triples, which is particularly suited for semantic web technologies. These backends are optimized for querying large sets of triples, enabling efficient access to the hierarchical and relational data in ontologies
2. **In-memory database**: Employed as a real-time data store, Redis is used in BioPortal for caching, session management, and as a message broker. Its use in BioPortal is geared towards reducing the load on the primary databases and speeding up response times. Redis is also used for handling temporary data that needs to be accessed quickly and is not critical if lost, such as session states or temporary computations.
3. **Search index**: Apache Solr is employed in BioPortal to index and search ontology data. Solr boosts the searchability of ontology terms by effectively indexing data stored in the triple-store databases. This allows for quick retrieval of data based on various search criteria, significantly improving the user experience when navigating through large sets of ontological graphs.
4. **Ontology artifacts and metadata storage**: The storage layer also handles the hosting of ontology artifacts, such as ontology files, and metadata associated with these ontologies. This involves maintaining versions of ontologies, tracking changes, and managing metadata that describes and contextualizes the ontological data.

#### BioPortal content and metadata

The ontologies in BioPortal are created by third parties, typically members of our user community. Ontologies are uploaded directly by registered users of the system, or derived from the quarterly release of the UMLS, or pulled automatically from the OBO Foundry. Every time a new ontology is added to the OBO Foundry, e.g., we manually upload it to BioPortal and ensure that we have consumed the ontology without errors. After that, our systems monitor the ontology daily and automatically re-upload it if it changes.

All content—including content derived from UMLS or OBO ontologies—is stored in a W3C standard ontology language, OWL or RDF, regardless of the original source format. These languages specify the use of Internationalized Resource Identifiers (IRIs) to uniquely identify ontology elements. Some of the ontology metadata are derived from author annotations to the ontology (e.g. provenance information), whereas other metadata are computed automatically by the system (e.g. metrics concerning ontology structure and ontology usage). If uploaded ontologies (and their component terms) are not already denoted by persistent identifiers, then BioPortal adds its own persistent URLs (PURLs) that are resolvable through a PURL service run at Stanford University.

### The BioPortal community

BioPortal is a freely available resource that anyone can use and contribute to. Users and developers are encouraged to contribute to BioPortal in multiple ways:

- Submitting an ontology;
- Adding comments to specific ontology terms;
- Adding mappings between ontology terms from different ontologies;
- Suggesting changes to an ontology (e.g. adding a new term, obsoleting a term, adding a new synonym, etc.);
- Reporting bugs or requesting features via the issue tracker in our open GitHub repository; and
- Submitting Pull Requests to improve the BioPortal code; these are evaluated and merged with our code base.

Sometimes developers in the community contribute major features to BioPortal. An example is the multilingual support functionality that was contributed to BioPortal by our partners at Le Laboratoire d’Informatique, de Robotique et de Microélectronique de Montpellier (LIRMM). Previously, BioPortal could only display terms in English, hindering the usability of ontologies for non-English-speaking users. Thanks to the developers at LIRMM, a new feature now allows users to switch between languages when viewing terms, making labels, descriptions, and other relevant information available in multiple languages.

We actively support the user and developer community of BioPortal through our dedicated support mailing list. On average, we handle ∼37 messages per month, addressing user inquiries, troubleshooting issues, and providing guidance on software usage and ontology-related topics. Our code is all publicly available on GitHub, and community members can submit bug reports, questions, and feature requests in our public issue tracker (https://github.com/ncbo/bioportal-project/issues), or suggest changes to the code base via Pull Requests.

### OntoPortal alliance

The success of BioPortal as a knowledge base of biomedical ontologies has stimulated interest from researchers in other disciplines. BioPortal is a web server, but it also includes a body of code that can be deployed independently and built upon. This has led to the formation of the OntoPortal alliance [12], which has developed the OntoPortal virtual appliance [13], building on the BioPortal infrastructure. The appliance can be installed behind firewalls at remote sites or adapted by users for their own domains or purposes. To date, we have recorded 273 deployments of the OntoPortal virtual appliance. Typically, these are sites that wish to use BioPortal with datasets that contain sensitive information such as protected health information that their users would not want to send over the internet, even in encrypted form. Over 1400 ontologies are available through several OntoPortal virtual appliances that address the needs of various scientific domains, including biomedicine (BioPortal), agriculture and agronomy (AgroPortal), ecology and biodiversity (EcoPortal), and ontologies in specific languages, such as biomedical content in Chinese (MedPortal).

Many members of the OntoPortal alliance community have contributed code to OntoPortal, enhancing the capabilities of the platform. This collaboration has expanded the pool of developers working on the OntoPortal code base, enabling the integration of improvements across different portals. There is a concerted effort among the alliance members to keep the code of individual members’ portals in sync with the global “OntoPortal” code base. This synchronization ensures that all enhancements and new features developed by any member benefit the entire community, maintaining a unified and robust infrastructure.

### Comparisons with related projects

BioPortal shares the biological and biomedical ontology space with several other prominent resources and platforms, including the OBO Foundry [1], the UMLS Metathesaurus [14], and the Ontology Look-up Service (OLS, https://www.ebi.ac.uk/ols4/). All of these platforms provide access to extensive collections of curated, hierarchical terminology. Some, like OBO Foundry, are primarily intended as registries rather than distinct resources and do not host downloadable ontology files or provide browsing or cross-ontology search capabilities. Others, like the OLS, are designed for searching and browsing ontology content, but are curated resources that are not intended to be open for submission; additional ontology portals such as Ontobee [15] and AberOWL [16] are aimed more at ontology researchers rather than the general community. Of these platforms, only BioPortal includes nearly all community-available ontologies in biomedicine while also allowing new ontologies to be kept private, if necessary. BioPortal has a quick, automated approval process for accepting submitted ontologies, so it is able to accept many more of them. It also offers more functionality, such as searching for terms across ontologies and requesting changes to ontologies, as described earlier. Crucially, BioPortal also includes all ontologies available through OBO Foundry as well as a wide selection of popular UMLS vocabularies, ranging from open-access sources to those with more restrictive licenses that limit usage to internal research, product development, and statistical analysis. See Table 2 for a summarized comparison between these platforms.

**Table 2.** Comparison of features distinguishing BioPortal from other ontology resources, platforms, and registries

Feature	BioPortal	OBO Foundry	UMLS	OLS
Indexes biological and biomedical ontologies	Yes	Yes	Yes	Yes
Includes all OBO ontologies	Yes	Yes	No	Yes
Includes UMLS ontologies (if license allows)	Yes	No	Yes	No
Provides downloadable files for ontologies (if license allows)	Yes	No	Yes	No
Provides a browsable interface for each ontology	Yes	No	No	Yes
Allows submission of private ontologies	Yes	No	No	No
All new ontologies are manually evaluated before inclusion	No	Yes	Yes	Yes
Supports searching for terms across ontologies	Yes	No	Yes	Yes
Supports annotating text with ontology terms	Yes	No	No	No
Includes automated textual mappings between terms	Yes	No	Yes	No
Supports semi-automated change requests	Yes	No	No	No

“OBO Foundry” refers to the Open Biological and Biomedical Ontology Foundry platform available at obofoundry.org. “UMLS” refers to the National Library of Medicine (NLM) Unified Medical Language System’s Metathesaurus, its biomedical thesaurus resource. OLS refers to the European Bioinformatics Institute (EBI) Ontology Look-up Service, version 4, available at www.ebi.ac.uk/ols4/.

## Future work

### Improvements to BioPortal capabilities

We will continue to improve access to submitted ontologies across OntoPortal resources. Ontology pages will gain more detail, allowing users to rapidly determine how appropriate each ontology is to their desired use case. We will expand integration of automated changes and KGCL-defined updates to give the community more ways to keep ontologies current. We will enhance BioPortal’s KG features by supporting in-depth graph-based search and integration with large language model (LLM)-powered AI agents (see below).

### LLM-driven ontology annotation

We have been investigating approaches driven by LLMs for transforming unstructured data into structured knowledge (such as KGs). BioPortal ontologies can be used as annotation sources for information extraction in the OntoGPT [17] software package; we are developing an approach for using relationships between BioPortal ontology classes to identify relationships within text. If an LLM-driven information extraction process identifies the term “fever” within a manuscript, e.g., it might normalize the term to its MeSH equivalent (http://purl.bioontology.org/ontology/MESH/D005334) and use the relationships within the BioPortal KG to determine that a broader term in the Human Phenotype Ontology would be “Abnormality of temperature regulation” (http://purl.obolibrary.org/obo/HP_0004370).

### Concept centric view

We have prototyped an application called concept centric view (CCV), which extracts concept-specific information from the BioPortal triple store and presents it to users through charts and graphs. CCV’s current UI leverages existing REST endpoints already integrated into BioPortal. These endpoints will be updated to query the KG, and a new UI will be developed using these REST APIs to provide user-friendly views into the KG.

### Internationalization

Many ontologies use ontological standards like OWL to embed international annotations about their terms. The label, definition, and note fields can be internationalized using semantic standards. The BioPortal team is incorporating code provided by the AgroPortal team to handle internationalized concepts for multilingual use. Future opportunities include internationalization of BioPortal UIs.

### Metadata

BioPortal captures a small collection of metadata about each ontology in the system, provided by the submitter of the ontology. To support the FAIR Principles, a much larger metadata specification needs to be implemented. BioPortal has been evaluating the fastest route to support detailed metadata such as that specified in the MOD [18] standard for ontology metadata.

### Enhanced SKOS support

BioPortal currently provides basic browsing and searching of SKOS [19] vocabularies. In response to user requests, such as from the NIH Rapid Acceleration of Diagnostics Data Hub project [20], the BioPortal team plans to incorporate AgroPortal’s recent enhancements representing SKOS vocabularies with first-class viewing and API features.

### Interoperable mappings

Plans for enhancing our support of mappings between terms from different ontologies include adopting a philosophy similar to our approach for ontologies—focusing on hosting and serving mappings rather than creating or editing them. We plan to enhance visualizations (introducing new views or highlighting mapping availability), filter mappings using metadata or scoring, enable bulk downloads, and ensure compliance with SSSOM (an ontology mapping standard [21]) for import and storage. These planned enhancements aim to position BioPortal as an interoperable mapping repository supporting the community’s evolving needs.

## Data Availability

The BioPortal web server is freely available at https://bioportal.bioontology.org. All software discussed in this paper is open access, and is freely available at https://github.com/ncbo and https://doi.org/10.5281/zenodo.15284916.

## Notes

Present address: University of California San Diego, La Jolla, CA 92093, United States
